# Moving from Medicaid Expansion Coverage to Medicare Can Be a Burdensome Transition: A Qualitative Study

**DOI:** 10.1007/s11606-025-09789-9

**Published:** 2025-09-02

**Authors:** Susan L. Hayes, Mary E. Byrnes, Wendy Furst, Erin Beathard, Renuka Tipirneni

**Affiliations:** 1 Department of Health Services, Policy & Practice, Brown University, School of Public Health, Providence, RI USA; 2 Department of Surgery, University of Michigan, Ann Arbor, MI USA; 3 Department of Internal Medicine, University of Michigan, Ann Arbor, MI USA; 4 Institute for Healthcare Policy & Innovation, University of Michigan, Ann Arbor, MI USA

**Keywords:** health insurance transitions, affordable care act, low income

## Abstract

**Background:**

The Affordable Care Act expanded Medicaid eligibility for low-income adults who are not Medicare eligible while leaving in place states’ more restrictive dual eligibility criteria. When Medicaid expansion enrollees turn 65 and transition to Medicare as their primary insurer, they may lose Medicaid and face higher premiums and out-of-pocket costs, yet there is little understanding of how older adults navigate this change in insurance programs.

**Objective:**

To investigate the experiences of Medicaid expansion enrollees who transitioned to Medicare coverage at age 65.

**Design:**

This is an interpretive descriptive qualitative study. Primary data were collected through semi-structured in-depth interviews conducted from April 2022 to February 2023. Data were analyzed in an iterative process, informed by interpretive description, a non-categorical qualitative methodology initially developed to address problems in applied settings.

**Participants:**

Participants had to be at least 65 years old, reside in Michigan, and have transitioned from Michigan’s Medicaid expansion coverage to Medicare (either traditional or Medicare Advantage) between 2016 and 2022.

**Main Outcomes and Measures:**

Descriptive themes of participant experiences navigating the transition from Medicaid expansion coverage to Medicare.

**Key Results:**

The study included 30 participants aged 65–71. A majority were women and White, with diverse educational attainment and geographic representation within Michigan. Nineteen participants were Medicare-only (without dual coverage). Three main themes were identified. This change in insurance was (1) unwanted; (2) complicated; and (3) for those without dual coverage, financially burdensome.

**Discussion:**

Our findings suggest a better alignment of Medicaid eligibility criteria before and after age 65, and access to unbiased Medicare enrollment guidance could help facilitate a smoother transition between these programs.

**Supplementary Information:**

The online version contains supplementary material available at 10.1007/s11606-025-09789-9.

## INTRODUCTION

Medicaid expansion under the Affordable Care Act (ACA) has demonstrated significant benefits to enrollees’ access to care, financial security, and health^1–3^, but this coverage comes with an expiration date. The ACA expanded Medicaid as a primary source of coverage for individuals *not*eligible for Medicare. People lose Medicaid when they turn 65 unless they qualify for the more stringent criteria that applies to individuals aged 65 and older^4^. Medicare is popular with beneficiaries, but the program’s premiums and out-of-pocket costs frequently represent a heavy cost burden, particularly compared to Medicaid’s minimal cost-sharing^5,6^. Enrolling in Medicare Advantage, as about half of beneficiaries now do, may result in lower premium and out-of-pocket costs compared to traditional Medicare but will likely still represent an increase compared to Medicaid expansion coverage. Like traditional Medicare enrollees, beneficiaries in Medicare Advantage plans owe Medicare Part B premiums ($185 a month for most beneficiaries in 2025), although some plans offer a "Part B premium reduction" as a benefit. Additionally, not all Medicare Advantage plans have $0 premiums, and they may have deductibles, copays, and coinsurance. Further, while Medicare Advantage plans, unlike traditional Medicare, have an out-of-pocket maximum, the amount in 2025 is $9350. Thus, even Medicare Advantage may pose affordability issues for beneficiaries coming from Medicaid expansion coverage.

Notably, eligibility criteria for Medicaid as a supplemental form of coverage to Medicare (dual eligibility) vary by state but are generally more restrictive than for Medicaid expansion coverage. In addition to lower income limits, most states also impose monetary asset limits for dual eligibility, which are not required for expansion coverage (see Supplemental Appendix A for more detail).

These more restrictive eligibility limits set Medicaid expansion enrollees on divergent paths when they turn 65: some lose Medicaid, others keep it as secondary coverage. The difference between having full Medicaid and not is stark: Full Medicaid benefits include coverage of Medicare premiums and cost-sharing for Medicare services as well as for “wrap-around” services not covered by Medicare, including long-term care and medical transportation (and in some states, dental, hearing and vision benefits).

The loss of Medicaid was temporarily postponed for Medicaid expansion enrollees who turned 65 and enrolled in Medicare while the continuous enrollment provision of the COVID-19 Public Health Emergency (PHE) was in effect (March 18, 2020-March 31, 2023). During this period, states were generally prohibited from dropping anyone from their Medicaid rolls, although in some cases they could transfer enrollees to other eligibility groups. As a result, unless a state automatically moved a Medicaid expansion enrollee to the Medicaid group for those age 65 or older, they were not disenrolled from Medicaid expansion coverage upon Medicare enrollment. While the practical effect of this is not entirely clear across states, it appears that at least in some states, Medicaid expansion coverage acted as a secondary payer, covering Medicare cost-sharing (whether traditional or Medicare Advantage) but not premiums. This may have influenced affected beneficiaries’ decisions about enrolling in traditional vs. Medicare Advantage, and while the PHE was ongoing, appears to have mitigated cost-sharing differences between the two types of Medicare coverage.

Examining the experiences of older adults who have moved from Medicaid expansion coverage to Medicare can enhance our understanding of this transition between these public health insurance programs and its effect on individual lives. More than a decade after Medicaid expansion first became a state option, there have been no published studies to our knowledge examining how current Medicare beneficiaries navigated this change in insurance, including how they seek health care and manage their health. In this study, we investigate the perspectives of older adults who moved from Medicaid expansion coverage to Medicare coverage at age 65, before and during the PHE.

## METHODS

This is an interpretive descriptive qualitative study—a non-categorical qualitative methodology initially developed to address problems in applied settings^7^. The approach identifies patterns as comprehensive evidence-based knowledge, informed by the perceptions and experiences of the group under investigation. As outlined below, sample selection and size were guided by the concept of information power—an alternative to “saturation” as a way of evaluating sufficient sample size for a qualitative study. The study was deemed exempt from review by the University of Michigan Institutional Review Board and by the Brown University Human Subject Protection Program and followed the Standards for Reporting Qualitative Research (SRQR) reporting guideline.

### Participant Recruitment

We utilized multiple strategies to reach individuals in Michigan who made the transition from Medicaid expansion coverage to either traditional Medicare or Medicare Advantage, including paid Facebook advertisements and flyers in local clinics and senior centers in Michigan (see Supplemental Appendix B for more detail). Participants had to be at least 65 years old, reside in Michigan, and have transitioned to either type of Medicare from Michigan’s Medicaid expansion program to be eligible for the study.

### Sample Selection

We emailed potential participants who completed a brief web-based screening questionnaire to confirm eligibility and interest in participating. In selecting the sample, we considered participants’ sociodemographic characteristics, dual enrollment status, and transition to Medicare primary coverage before vs. during the PHE. We did not select based on participants’ Medicare coverage type (traditional or Medicare Advantage) as comparing participants’ experiences in traditional Medicare versus Medicare Advantage after enrollment was not a focus of this study. Rather, our study aim was to understand the experience of the coverage transition itself and, to the extent it varied, to compare experiences based on losing full Medicaid benefits or keeping them as secondary coverage (i.e., dual enrollment).

Our sample size was guided by the principles of information power, which considers the scope of the study aim, existing theory, quality of dialogue, analytic strategy, and specificity of the sample^8^. Our focus was relatively narrow in scope, and existing theory about how people experience the Medicaid-to-Medicare transition is lacking, indicating our findings had the potential to contribute new information. Further, the quality of the interview dialogue was high (i.e., study participants were able to talk at length and in great detail about their experiences making the coverage transition, producing rich data) and our group-based consensus inductive approach to analysis allowed the collected data to be examined in various capacities. In addition, our analytic strategy was rigorous and included coding, theme reports, biweekly discussion of theme reports with the full study team, and the development of thematic matrices and memos. Lastly, to be selected for the sample, individuals had to have direct personal experience making the transition from Medicaid expansion coverage to Medicare in Michigan at age 65. While this narrowed considerably the group from which to recruit, it also ensured a highly specific and knowledgeable sample. These factors combined with the attention paid to dual status, transition timing, and sociodemographic characteristics during sample selection allowed us to feel confident that our sample satisfied the criteria of information power and captured a wide variety of experiences.

### Qualitative Interview Procedures

Interviews were conducted from April 2022 to February 2023 by a single team member (S.H.) via telephone and secure video platform. All participants verbally consented to and participated in a 50- to 90-min interview. Interviews were semi-structured and included questions about: experiences with Medicaid expansion coverage prior to turning 65; navigating the Medicaid expansion coverage to Medicare transition including Medicare enrollment and applying for dual eligibility; differences in costs and covered benefits under Medicare (whether Medicare-only or dually enrolled in Medicare and Medicaid) compared to Medicaid expansion coverage, including perceived affordability; and any differences participants experienced in obtaining health care after making the transition to Medicare from Medicaid expansion coverage. (See Supplemental Appendix C for Interview Guide.) Interviews were recorded and transcribed verbatim by an external transcriptionist who redacted identifying information, and transcriptions were reviewed for accuracy. Participants who completed the interview were mailed a $25 gift card.

### Data Analysis

Interviews from all participants were analyzed through an iterative process and informed by interpretive description (ID), a flexible qualitative methodological approach developed by Thorne and colleagues. What distinguishes ID from other qualitative research approaches is its focus on producing insights and practical knowledge that can then be applied in real-world settings such as health care and education. While qualitative methods such as phenomenology and grounded theory emphasize, respectively, the exploration of individual experiences and the generation of new theories, ID, by contrast, is geared toward helping to improve policy and practice. One of the key strengths of ID is that it values the researcher’s own background knowledge of and professional experience with the phenomenon under investigation, viewing topic familiarity as an advantage as opposed to something to conceal^9^.

All members of the study team reviewed several transcripts to identify patterns and commonalities in the data. We then developed an initial codebook based on team members’ individual readings of the transcripts. Two members from our study team (S.H. and W.F.) tested the codebook on three interview transcripts, and we met regularly as a team to discuss coding strategy and develop new codes to capture further commonalities observed in the data. Coded data were prepared into a data matrix (S.H. and W.F.), which the study team reviewed and discussed to identify initial themes. Themes were developed and refined through the review and discussion of written theme reports, with attention paid to determining whether themes were “universal” (i.e., reflected the experiences of participants regardless of dual enrollment status or timing of Medicare entry) or group specific. Data were coded and managed using MaxQDA20 (Verbi Software, Berlin, Germany).

Steps taken to ensure reflexivity and rigor included a diverse study team with divergent backgrounds and perspectives and the use of critical dialogue as well as our iterative analytic strategy. Our research team brought a broad mix of professional training and personal experiences to this study, which shaped our methods of gathering, analyzing, and interpreting data. We represented a range of racial, economic, and educational backgrounds, which helped us engage with the research topic from multiple angles. Recognizing that our identities and social positions could influence how we viewed the data, we regularly paused to reflect on how our perspectives might shape our interpretations. Throughout the research process, we remained committed to elevating the voices of participants and ensuring that our findings reflect their real transitional experiences.

## RESULTS

The study included 30 participants aged 65–71. A majority were women and White, with diverse educational attainment and geographic representation within Michigan. Nineteen participants were Medicare-only (without dual coverage), nine had Medicare-Medicaid, and two were enrolled in a Medicare Savings Program that paid their Part B premiums. Most enrolled in Medicare during the PHE (Table 1).

**Table 1:** Participant Demographic Characteristics (*n* = 30)

Characteristics	Number of participants
Female	19
**Race, ethnicity**	
Asian	1
Black/African American, Non-Hispanic	3
Native American/Alaskan Native	2
White, Non-Hispanic	24
**Geographic region in Michigan**	
Upper Peninsula/Northwest/Northeast	3
West/East Central/East	6
South Central/Southwest/Southeast	15
Detroit Metro	6
**Educational attainment**	
High school diploma	5
Some college	9
Associate’s degree	4
Bachelor’s degree	8
Post-graduate degree	4
**Medicare enrollment**	
Enrolled in Medicare Before COVID-19 Public Health Emergency (PHE)	9
During PHE (2020–2022)	21
**Medicare coverage**	
Medicare only, no Medicaid	19
Medicare + full Medicaid benefits	9
Medicare Savings Program, without full Medicaid	2
**Low-income subsidy for Medicare Part D prescription drug costs**	
Yes	19
No	9
Not sure	2

We identified three main themes that describe the experiences and challenges of our sample as they transitioned from Medicaid expansion to Medicare coverage. This change in insurance was (1) unwanted, given high satisfaction with Medicaid expansion coverage; (2) complicated, given Medicare’s more complex enrollment process compared to Medicaid expansion coverage and different requirements for Medicaid as supplemental vs. primary coverage; and (3) for those without dual coverage, financially burdensome.

### Theme 1: An Unwanted Transition

Participants were overwhelmingly positive about the Medicaid expansion coverage they had immediately prior to turning 65, whether they transitioned to have dual coverage or not (Table 2). The majority lauded the comprehensiveness of the coverage, provider networks, quality of care, and especially, the minimal costs. A participant elaborated, “I loved the fact that there was no premium. There was no copay. No coinsurance. It was real insurance. It took care of you and your needs.” Some participants judged Medicaid to be better than the private insurance they had previously. “I had good insurance through work. But Medicaid is the best insurance coverage I have ever had,” one enthused.

**Table 2:** Participant Perspectives on an Unwanted Transition

I was so sad when Medicaid left me. (Female, White, Medicare-only)
Never once did I have any problems at all [with Medicaid expansion coverage]. I’m telling you, it was great! (Male, White, Dual Eligible)
Turn[ing] 65 and be[ing] switched to Medicare, might be a savings to most, because maybe they get better coverage than what they had with… their employer [or] whatever. For most people that would be a pleasant transition. For anyone that was on Medicaid, like myself, not at all. There was nothing to be gained by it. (Male, White, Medicare-only)
[Medicaid expansion coverage] offered a lot of other things that you needed. It offered dental care. It offered care for vision and hearing. Those things are not offered under Medicare. (Female, Black, Medicare-only)
I can’t believe that it was actually better under [Medicaid expansion coverage] than it was under Medicare. Because…it’s supposed to be a benefit to senior citizens, many of whom have been paying into it for a long time. (Male, White, Medicare-only)
What was my criteria for picking [Medicare Advantage Dual plan]?… I wanted to stay with the group of doctors that I was working with. It is a comfort to me because, you know, there is a relationship. If I walk in the office of a doctor who is unfamiliar to me and I had to start over…. (Female, White, Dual Eligible)
I was kind of…if Medicaid would pay for everything, why do I have to have Medicare now? Why can’t I just stay on Medicaid? (Laugh) But it doesn’t work that way. (Female, White, Medicare-only)

Given their high level of satisfaction with Medicaid expansion coverage, participants were not eager to change their insurance when they turned 65. A participant explained, “I thought, ‘Medicaid is great. I’ll just stay on that.’ But they told me at DHHS that you have to apply for Medicare.” Another questioned, “Why make people change something that’s already working good? Especially if everything is the same, why make them change?”

Participants acknowledged that Medicare was similar to, if not better than, Medicaid expansion coverage in at least one respect. They were able to keep the same providers and have access to a broader choice of providers. A dual participant observed “so far, [my doctors] have all taken what I have, the Medicare and Medicaid…. It is just that it seems like there are a few more doctors that accept it than [the state’s Medicaid expansion coverage].” However, for participants without dual coverage, that continuity and greater choice came at a price. As one noted, “My healthcare has not changed because I was able to keep the same doctors. And that’s fine. It’s just you are paying more for the same thing.” Neither dual nor non-dual participants sought this coverage change, but after the transition was complete, dual participants were generally content, while non-duals were averse.

### Theme 2: A Complicated Transition

Many participants reported that the difference between Medicaid expansion coverage and Medicare became apparent as soon as they began the Medicare enrollment process (Table 3). While individuals who are already receiving Social Security benefits before they turn 65 are automatically enrolled in Medicare Part A (hospital insurance) and Part B (medical insurance), those who are not must enroll through the Social Security Administration. Beneficiaries who want Part C (Medicare Advantage) or Part D (prescription drug coverage) must choose a plan and enroll in it. Few participants, whether dual or non-dual, were prepared for the complexity of enrolling in Medicare, with its multiple parts and various levels of financial assistance. Some contrasted the process with that of applying for Medicaid expansion coverage: “I thought Medicare was Medicare. I remember on Medicaid, I didn’t have to do anything, but [after enrolling in Medicare Part B] I find out I now have to apply for prescription coverage. Then I found out that I have to apply at DHS again, this time to have the [Part B premium] covered.”

**Table 3:** Participant Perspectives on a Complicated Transition

Signing up for Medicare was a lot harder than Medicaid because they had Parts A and B and C and D, and you had to make sure you get [the financial assistance with Medicare costs] you are entitled to. (Female, White, Dual Eligible)
[The state] started telling me you’ve got to apply for Medicare. So, I started reading up on it, and googling everything and making lots and lots of phone calls….I had to call many times. Not because they were bad but because it’s complicated. (Female, White, Medicare-only)
I got an email that I didn’t qualify for Medicaid expansion coverage anymore, which is true, [but] they did not offer any information. In that regard, the transition wasn’t that easy. I had to do research on my own [to find out] I had to apply for a different program. (Male, White, Dual Eligible)
They say pick out the [Part D] plan that best works for you. You get cross-eyed looking at all of these. If I pick this, it covers this and this and this. But it doesn’t cover this. And then you go down to the next one. And it’s like, forget it. This is what they [automatically] enrolled me in. I [will] just take it. (Female, White, Dual Eligible)
To me, they should be saying, `Well, okay, how much assets?…I’m a single person. I’m divorced. I don’t have anybody else’s income helping me out. But they don’t consider that. (Female, White, Medicare-only)
I thought, ‘Well if I’m approved for [the Low Income Subsidy], then I will be able to at least get help paying for my Medicare [premiums],’ And I applied. And nope, at $19,000 a year I was over income. (Female, White, Medicare-only)

All participants commented on the deluge of mail, phone calls, and targeted advertising they received from Medicare plans when they turned 65. Several participants found the marketing blitz predatory, and it left them unsure about whom they could trust to provide unbiased information. As one said, “You get all these people calling you… ‘We’ll help you. We’ll help you.’ And unless you know who you want to talk to or who you want to trust, you’re taken by anybody.” This led numerous participants to research health plans on their own, a task they described as time-consuming and challenging due to the difficulty in making head-to-head comparisons of plans. “They compare apples to oranges, and then say that you need to eat fruit cocktail,” one quipped.

Several participants said it was only through the intervention of a friend, relative, or Social Security Administration worker that they were able to successfully navigate the enrollment process. A participant gratefully recounted the assistance they received from a worker at the local Social Security office: “I must have looked like a scared rabbit because [the worker] said, ‘You know what? I’m going to get all the paperwork filled out for you.’”

The frustration was compounded for participants who reported they did not qualify for dual coverage. They described being caught off guard by the more restrictive financial eligibility requirements for Medicaid as supplemental insurance compared to Medicaid expansion coverage. “I tried to get…Medicare and Medicaid together. And then the caseworker looked at my income. And she said that I was not qualified.” Non-dual participants perceived the differing requirements for Medicaid before and after age 65 as unfair: “When I hit whatever magic age it was, when I could collect Medicare, they pulled the rug out from under me.” Several described applying for a Medicare Savings Program that would cover their Part B premiums only to be turned down because their income was over the eligibility cut-off, sometimes by the slimmest of margins (e.g., $10).

The asset test for Medicaid as a form of supplemental coverage vexed non-dual participants even more. They criticized the inconsistency between ignoring monetary assets when applying for Medicaid expansion coverage and invoking them as a disqualification for Medicaid supplemental coverage. “For the Medicaid [expansion coverage], I always tell them, ‘Look. I have this annuity. I have this annuity.’ And they’re like, ‘That doesn’t matter. That doesn’t matter.’…I tried to get on the [Medicare Savings Program]. I don’t qualify because I have an annuity! Even though I don’t draw anything from it yet.” As with the income cut-off, affected participants reported it felt unfair that the margin by which assets exceed the limit is not considered, nor are the nuances and complexities of people’s lives that can affect retirement income and savings, such as divorce and unpaid family caregiving.

Further confusion around dual eligibility emerged during the PHE. While several participants who enrolled in Medicare during the PHE said they eventually surmised that their Medicaid expansion coverage was acting as a secondary insurer because they did not have cost-sharing, they reported this was not communicated to them. Some believed that because they still had Medicaid coverage due to the continuous coverage provision, it meant they were dual eligible, only to later find out otherwise. A participant who postponed taking Social Security benefits and therefore needed to pay Part B premiums to Medicare directly assumed the state was paying the premiums as it does for dual-eligible beneficiaries—until the bill arrived for the unpaid months of premiums.

### Theme 3: A Financially Burdensome Transition without Dual Coverage

For participants without dual coverage, the challenges of navigating Medicare enrollment were surpassed by the sticker shock they experienced at their suddenly higher health care costs (Table 4). Whether they opted for traditional Medicare or Medicare Advantage, non-dual participants went from owing no or modest premiums for Medicaid expansion coverage to owing, at a minimum, the standard monthly premium for Medicare Part B. One participant, who made the transition to Medicare in 2022 when the monthly premium was $170.10, described the change, “I mean, the whole $170 just kicked me like a ton of bricks. I thought I’d have to pay nothing to Medicare.”

**Table 4:** Participant Perspectives on a Financially Burdensome Transition Without Dual Coverage

Yeah, I do have to give up [things]. I don’t use my air conditioner unless it is a horrible day. I don’t use water. I conserve in all of my utilities…I’ve had to restrict my buying. Everything. Just so I can [afford my premiums]. (Female, White, Medicare-only)
They [participant’s Medicare Advantage Plan] don’t cover [medications] that my doctor wants me on. One that I’m supposed to take, that’s like $1200–1400 a month. That one was covered 100 percent on Medicaid. So, I’m not taking it. (Female, White, Medicare-only)
They wanted me to go through rehab [after surgery]. Rehab was like $50 per shot and they said you were going to go three times a week….I said, ‘what is involved?’…I did that on my own [at home]. (Male, White, Medicare-only)
This is the part that scares me, if I did have a hospital stay, if I did have a cancer diagnosis. I know that a lot of services would be used, and I don’t know how much my responsibility would be. With the Healthy Michigan Plan [Michigan’s Medicaid expansion coverage], I knew that I wouldn’t have any responsibility afterwards. It was always 100 percent covered. (Female, White, Medicare-only)
I understand now where seniors [who] don’t have a Medicaid backup.…You hear horror stories, [choosing] between meds and food. And it’s like, I understand that now. (Female, White, Dual Eligible)
I’m a happy camper. If something goes wrong, and I’m not paying for it, I’m happy. (Male, White, Dual Eligible)

Several Medicare-only participants reported they now spent as much as $300 to $400 monthly on premiums. While that may be more typical with traditional Medicare than Medicare Advantage, the Part B premium still makes up the largest share. One participant with a Medigap and standalone Part D plan broke it down: “I am paying the $170.10 [for Part B in 2022]. I’m paying $128.08 for the Part G [Medigap plan]. I’m paying $12.70 for the Part D. And I’m paying $16.34 for the vision.”

Medicare-only participants also faced increased cost-sharing compared to Medicaid expansion coverage. One participant enumerated the difference in their out-of-pocket costs for prescription drugs: “[Prescription copays] were zero with Medicaid. [Now] one medication every six months, I pay $400 for. So, that’s $800 a year for one medication. The rest of them are probably $50 a month. So, add that, there’s $1,400 a year.”

Nearly all participants who were not dual eligible said they made financial trade-offs to afford their Medicare premiums and out-of-pocket costs, including continuing to work or returning to the workforce. Several participants detailed sacrifices they made to pay for their health care expenses, including selling possessions, cutting out discretionary expenses such as travel, restaurant meals, and new clothing purchases, putting off home repairs, canceling internet and cable services, and sparingly using utilities. A few participants mentioned that they relied on food banks to get enough to eat.

Even with this belt-tightening, several Medicare-only participants reported delaying or going without needed health care because of the costs. A participant who made the transition to Medicare before the PHE (and therefore did not have a temporary reprieve from Medicare cost-sharing) delayed visiting a specialist: “I had some lab tests done, and they think I have lupus. I would have to go to a rheumatologist…and honestly, I can’t afford the copays to see a rheumatologist.” Others also worried about how they would afford hospital care, a concern they did not have with Medicaid expansion coverage. One elaborated, “I have had cancer and I had a hernia. I had a neck operation. I didn’t pay a dime for any of that when I was on Medicaid. I am scared now, if I had to go into the hospital what it would end up costing me.”

Moreover, some participants who enrolled in Medicare during the PHE and who expected to lose Medicaid during the “unwinding” of the Medicaid continuous enrollment provision were bracing themselves for the higher out-of-pocket costs that will inevitably follow. “[When] my [Medicaid expansion] coverage is up for renewal, that’s probably when I’m going to get kicked off. I’ll have to pay all the [Medicare] deductibles and all the copays. So yeah, I get to work more!” Further, many Medicare-only participants reported anxiety about continuing to afford their health care costs in the future, especially when they can no longer make the sacrifices they are making now. A Medicare-only participant with multiple chronic conditions explained: “I feel like I am walking on thin ice. I can only assume that eventually I am going to end up back on Medicaid because I will be unable to function.”

These affordability concerns, however, represent one side of a gulf. On the other side lies the experiences of those participants who qualified for dual coverage. One described the combination: “What Medicare doesn’t cover, Medicaid will. Even vision and dental, I don’t have a copay.” Another participant tallied their health care expenses as a dual-eligible beneficiary: “I think my monthly medical bill may max out at $5.” No participant with full Medicaid benefits in addition to Medicare recalled going without care because of cost concerns. But they acknowledged that without dual coverage, the scenario would likely be different: “I probably would [go] without some of my health care.” Another, who had suffered a cardiac arrest and a stroke, and had been in a coma and on a ventilator, affirmed that without the combined coverage of Medicare and Medicaid they would be saddled with medical debt.

## DISCUSSION

This qualitative study provides greater understanding of how older adults with low incomes who gained Medicaid expansion coverage under the ACA navigate the transition to Medicare as their primary insurer. It also offers insights into how this change in health insurance coverage affects individual lives. Participants reported a high level of satisfaction with Medicaid expansion coverage and a reluctance to leave it for another form of insurance. At the same time, they appreciated the continuity of patient-provider relationships and access to expanded networks of providers under Medicare. This is noteworthy because continuity of care by the same providers is associated with higher patient satisfaction, increased take-up of preventive care services, improved medication adherence, and fewer ED visits and hospitalizations^10^.

However, this positive aspect was largely overshadowed by participant frustration with the complexity of the Medicare enrollment process, made more confusing during the COVID-19 PHE—and for those without dual coverage, consternation over the more restrictive eligibility criteria for Medicaid as supplemental coverage. For several participants, the frustration of the Medicare enrollment process was compounded when they learned they did not qualify for dual eligibility. Some participants who reported not qualifying for dual eligibility said their income exceeded the eligibility limit by only a few dollars. This may be a relatively common experience in the dozen Medicaid expansion states including Michigan that cap income eligibility for full Medicaid at 100% FPL and for a Medicare Savings Program (MSP) that covers Part B premiums at 135% FPL (for a single person)—a mere 3 percentage points lower than it is for Medicaid expansion coverage, or a difference of about $38 per month in 2024. An additional 22 expansion states have the same MSP income limit.

Not qualifying for dual eligibility can be associated with a staggering jump in premium and out-of-pocket costs for former Medicaid expansion enrollees. Participants described going from minimal or no costs for Medicaid expansion coverage to paying as much as $300 to $400 a month for Medicare, in premiums alone. Deductibles, copayments, and coinsurance often add up to hundreds (in some cases, thousands) of dollars annually. To afford these expenses, participants reported making difficult trade-offs, including going back to work, forgoing medical care or prescribed medications, and making deep cuts to their personal budget. They expressed anxiety about their ability to maintain these sacrifices and the potential consequences when they no longer can.

Ironically, the success of the ACA’s Medicaid expansion may alter the view of Medicare as a beacon among lower-income older adults. Prior to the ACA, turning 65 brought with it the certainty of health care coverage and access to care, especially for older adults with low incomes who didn’t have access to job-based insurance. Non-disabled adults without children rarely qualified for Medicaid coverage, and premiums for private insurance were often prohibitively high for this age group—if a policy could be purchased at all. A 2007 survey by the Commonwealth Fund found that among adults aged 50–64 who had bought, or tried to buy, an individual health insurance policy, nearly two in five (39%) reported being denied coverage, charged a higher price, or had treatment for a pre-existing condition excluded^11^. Studies conducted prior to the ACA provided evidence that gaining Medicare coverage led to positive health outcomes. McWilliams and colleagues found that older adults with common chronic diseases who had been uninsured prior to age 65 used more health services in their initial years of Medicare coverage than their counterparts who had been continuously insured and had greater improvements in health outcomes^12,13^. Among adults with emergency hospital admissions who had just turned, or were about to turn, age 65, Card, Dobkin, and Maestas found that patients who had recently turned 65 received more services during their hospital stay, were more often transferred to another unit in the hospital such as a skilled nursing facility before being discharged, and had reduced rates of readmission and mortality compared to their 64-year-old counterparts^14^.

Since the ACA’s coverage expansions and insurance reforms, however, adults nearing Medicare eligibility age are less likely to be uninsured and may have more comprehensive coverage than their predecessors—especially in the 40 states that, along with the District of Columbia, have now adopted the Medicaid expansion. Middle-aged adults with Medicaid expansion coverage now have access to care that addresses acute and chronic health concerns and the peace of mind that comes with knowing such access is affordable. In this context, a transition to Medicare may be less appealing. It brings a more confusing enrollment process, and for those who do not meet the more restrictive financial requirements for dual eligibility, higher premium and out-of-pocket costs and anxiety about affording health care costs in the future. For Medicaid expansion enrollees, gaining Medicare as a primary insurer may be more a bane than a boon if they lose Medicaid coverage in the process.

### Limitations

As one of the first studies to assess older adults’ experiences of entering Medicare in the post-ACA era, our study begins to fill an important gap. It does have several limitations, however. First, dual enrollment status is self-reported, although the specificity with which participants discussed applying for and being approved or denied dual coverage inspires confidence in the accuracy of their self-assessment. Second, our findings may not be fully generalizable to states with different income and asset levels for Medicaid supplemental coverage and MSPs or to states with alternative Medicaid expansion models. Third, most participants made the transition to Medicare while the continuous Medicaid enrollment provision of the PHE was in effect. However, this timing only heightens the relevance of our finding that non-dual participants struggled to afford Medicare expenses. Without Medicaid expansion coverage to act as a secondary payer, those expenses will be greater.

Additionally, the sample was predominantly White and female, though it did include people who identified with diverse sociodemographic characteristics. We found, however, differences in perspectives to be most sharply drawn based on dual eligibility status as opposed to race, ethnicity, gender, or educational attainment. Lastly, the findings are not differentiated by type of Medicare enrollment (traditional or Medicare Advantage). Our study focused on the transition to Medicare itself, as both forms of Medicare coverage can differ from Medicaid expansion coverage in terms of enrollment processes, premium and cost-sharing requirements, and provider networks. However, the unique circumstances of the PHE’s continuous enrollment provision, which affected the majority of our sample and coincided with our data collection period, would have complicated any attempt to meaningfully compare experiences between the two Medicare enrollment types. Future work that does so would be a high priority.

## Conclusions

In this qualitative study, older adults who transitioned from Medicaid expansion coverage to Medicare at age 65 expressed frustration with the complexity of the Medicare enrollment process. For those who did not have dual coverage, this was compounded by differing requirements for Medicaid before and after age 65 and the need to make difficult and potentially unsustainable trade-offs to afford Medicare premiums and cost-sharing. Better alignment of Medicaid eligibility criteria and improved access to unbiased Medicare enrollment guidance could go a long way in easing Medicaid to Medicare transitions.

## Supplementary Information

Below is the link to the electronic supplementary material.

Supplementary file1 (DOCX 30.5 KB)

## Data Availability

The interview data from this study are not available to protect participant confidentiality.

## References

[1] **Miller S, Wherry LR.** Health and access to care during the first 2 years of the ACA Medicaid expansions. *N Engl J Med*. 2017;376(10):947-56. 10.1056/NEJMsa1612890 28273021 10.1056/NEJMsa1612890

[2] **Hu L, Kaestner R, Mazumder B, Miller S, Wong A.** The effect of the Affordable Care Act Medicaid expansions on financial wellbeing. *J Public Econ*. 2018;163:99-112. 10.1016/j.jpubeco.2018.04.009 30393411 10.1016/j.jpubeco.2018.04.009 PMC6208351

[3] **Sommers BD, Maylone B, Blendon RJ, Orav EJ, Epstein AM.** Three-year impacts of the Affordable Care Act: improved medical care and health among low-income adults. *Health Aff* (Millwood). 2017;36(6):1119-28. 10.1377/hlthaff.2017.0293 28515140 10.1377/hlthaff.2017.0293

[4] **Ndumele CD, Sommers BD, Trivedi AN.** The ACA’s 65th birthday challenge: moving from Medicaid to Medicare. *J Gen Inter Med*. 2015;30(11):1704-6. 10.1007/s11606-015-3328-0 10.1007/s11606-015-3328-0 PMC4617934 25925342

[5] **Schoen C, Davis K, Willink A.** Medicare beneficiaries’ high out-of-pocket costs: cost burdens by income and health status. Commonwealth Fund. Available at: https://www.commonwealthfund.org/publications/issue-briefs/2017/may/Medicare-beneficiaries-high-out-pocket-costs-cost-burdens-income. Accessed June 24, 2024. 28498650

[6] **Madden, JM, Bayapureddy, S, Briesacher, BA, Zhang, F, Ross-Degnan, D, Soumerai, SB, Gurwitz, JH, & Galbraith, AA.** Affordability of medical care among Medicare enrollees. *JAMA Health Forum*. 2021;2(12):e214104. 10.1001/jamahealthforum.2021.4104 35977305 10.1001/jamahealthforum.2021.4104 PMC8796945

[7] **Thorne S.** *Interpretive description: qualitative research for applied practice*. Milton Park: Routledge; 2016.

[8] **Malterud K, Siersma VD, Guassora AD.** Sample size in qualitative interview studies: guided by information power. *Qual Health Res*. 2016;26(13):1753-60. 10.1177/1049732315617444 26613970 10.1177/1049732315617444

[9] **Thompson Burdine J, Thorne S, Sandhu G.** Interpretive description: A flexible qualitative methodology for medical education research. *Med Educ*. 2021;55(3):336-43. 10.1111/medu.14380 32967042 10.1111/medu.14380

[10] **Gray DJP, Sidaway-Lee K, White E, Thorne A, Evans PH.** Continuity of care with doctors—a matter of life and death? A systematic review of continuity of care and mortality. *BMJ Open*. 2018;8(6):e021161. 10.1136/bmjopen-2017-021161 10.1136/bmjopen-2017-021161 PMC6042583 29959146

[11] **Collins SR, Doty MM, Garber T.** Realizing health reform’s potential: adults ages 50–64 and the Affordable Care Act of 2010. Commonwealth Fund. Available at: https://www.commonwealthfund.org/publications/issue-briefs/2010/dec/realizing-health-reforms-potential-adults-ages-50-64-and, Accessed June 24, 2024. 21155372

[12] **McWilliams JM, Meara E, Zaslavsky AM, Ayanian JZ.** Use of health services by previously uninsured Medicare beneficiaries. *NEJM*. 2007;357(2):143–153. 10.1056/NEJMsa067712 10.1056/NEJMsa067712 17625126

[13] **McWilliams JM, Meara E, Zaslavsky AM, Ayanian JZ.** Health of previously uninsured adults after acquiring Medicare coverage. *JAMA*. 2007;298(24):2886-94. 10.1001/jama.298.24.2886 18159058 10.1001/jama.298.24.2886

[14] **Card D, Dobkin C, Maestas N.** Does Medicare save lives? *Q J Econ*. 2009;124(2):597-636. 10.1162/qjec.2009.124.2.597 19920880 10.1162/qjec.2009.124.2.597 PMC2777733

