# Supplementary material for: Moving from Medicaid Expansion Coverage to Medicare Can Be a Burdensome Transition: A Qualitative Study
Source: J Gen Intern Med. 2025 Sep 2;41(5):1241–9. doi: 10.1007/s11606-025-09789-9 (PMC12908127; doi:10.1007/s11606-025-09789-9)
Supplement: Supplementary file 1 — Supplementary file1 (DOCX 30.5 KB) [file 11606_2025_9789_MOESM1_ESM.docx]

**Supplemental Appendix**

**Appendix A: Detailed Background**

*Dual (Medicare/Medicaid) eligibility criteria relative to Medicaid expansion coverage*

Dual eligibility criteria vary by state, but nearly half of states cap older adults’ Medicaid income eligibility at 74% of the federal poverty level (FPL), roughly half the limit for expansion coverage.^1^ Most states also impose monetary asset limits of $2,000 (individual) /$3,000 (couple), which are not required for expansion coverage.^1^ California and New York are exceptions, having effectively raised their income limit for dual eligibility to 138% FPL, the same as for expansion coverage, and significantly increased or eliminated asset limits.^2-4^

Medicare Savings Programs (MSPs)—sometimes called partial Medicaid—pay for Medicare Part B premiums and, for people living at or below the poverty line, cost sharing for Medicare-covered services. MSPs have higher income limits but still lower than for Medicaid expansion coverage in most states; additionally, 36 states have asset tests for these programs.^1^

*Policy Context*

Michigan’s Medicaid expansion program, called the Healthy Michigan Plan (HMP), began on April 1, 2014. Prior to January 2024, HMP enrollees with incomes between 100-138% FPL (including the allowed 5% income disregard) had monthly fees equivalent to 2% of household income, and all enrollees (except for certain exempt groups) could be charged modest co-pays based on their income and type of health services received. Total cost sharing was capped at 5% of annual household income. There were 115,531 HMP enrollees age 55 to 64 as of May 27, 2025, constituting 16% of total enrollment in HMP.^5^

**Appendix B: Detailed Methods**

*Participant Recruitment*

We utilized three strategies to reach individuals in Michigan who made the transition from Medicaid expansion coverage to Medicare. First, we launched a paid targeted social media advertising campaign on Facebook between April and December 2022, which was targeted to individuals who 1) resided in Michigan and 2) were age 65 or older, and 3) whose Medicare eligibility was based on age. Second, we publicized the study on the University of Michigan’s Health Research web site that connects patients and community members with university research studies. Third, we created a recruitment flyer distributed to the state’s 40 Federally Qualified Health Centers for posting or distribution at their 160 sites, a University of Michigan general internal medicine clinic, and a local Senior Center.

**Appendix C: Interview Guide**

**Part 1. Introduction**

Thank you for agreeing to talk with us. We are part of a research team at the University of Michigan and at Brown University trying to understand how people like you experience changes in their health insurance when they turn 65. We are very interested in how people experience changes from Medicaid to Medicare insurance.

- We are speaking with people who are at least 65 years old, who are eligible for Medicare insurance now but who had Medicaid insurance under the Affordable Care Act’s Medicaid expansion before enrolling in Medicare.
- The interview takes about 45 to 60 minutes and includes questions about you, your experiences with your health insurance, and accessing health care.
- Participating in the interview is voluntary. You don’t have to answer any question you don’t want to. You can decide to end the interview at any time.
- We can take a break whenever you want. You can also ask us questions at any time.
- Your answers will be kept confidential. Your information will be stored at the University of Michigan in a secured computer file that does not include your name.
- Reports will combine information from many people who are participating in this project; individuals will not be identified in any reports. We will not tell the Medicaid or Medicare programs or your doctors any of the answers you give.
- For completing the interview, you will get a $25 gift card.

Do you have any questions before we begin?

We would like to audio record our conversation, so I can ensure the completeness and accuracy of our conversation. We will not share the recording with anyone besides the research team. We will turn off the audio recorder at any time at your request. Would that be ok with you?

**[If YES, BEGIN RECORDING]**

[If NO, verify that recorder is turned off and then take notes.]

Ok, let’s get started.

1. Before we start talking about your experiences with Medicaid and Medicare, I’d love to learn a little more about you. Whatever you feel like sharing about yourself would be interesting to me but maybe you could tell me about where you live in Michigan and what kind of work you do or have done there.

**Part 2. Experiences with Medicaid Expansion Coverage Before Turning 65**Now turning to experiences with your health insurance. I know you have Medicare insurance now but thinking back to the time **before** you had Medicare, when you had Medicaid coverage…

1. Tell me about how long you had Medicaid coverage before getting Medicare.
2. As you know for this study, we are talking with people who had Medicaid coverage through the Healthy Michigan Plan. Tell about how you found out about this coverage and enrolled in it?
3. Tell me about what it was like to get health care when you had Medicaid coverage.  *Probes:*

- Where did you go to get health care?
- Did you have a personal doctor—that is, were you able to go to the same doctor or practice whenever you needed care?
- Was it easy or difficult to find a doctor or clinic that took your Medicaid insurance?

**Follow up:** Tell me about how you found your doctor (or clinic).

- Did you ever need to see any specialists when you had Medicaid? By specialist, I mean a medical provider who specializes in one area of care, such as a heart doctor or an allergy doctor.
  **Follow-up:** If yes, tell me about that experience.
  *Sub-Probes:*
- Was it easy or difficult to find a specialist who took your Medicaid insurance?
- How did you go about finding a specialist?
- And what about getting medications that your doctor prescribed for you when you had Medicaid? Tell me about what that was like.
  *Sub-Probes:*
  - - Were there ever any instances when your doctor prescribed a medication and the pharmacy said your insurance wouldn’t cover it? If so, tell me about that.

1. Now let’s talk about your health care costs when you had Medicaid coverage. Tell me about any expenses you had to pay for health care when you had Medicaid. *Probes:*

- Did you have to pay a monthly fee for your Medicaid coverage—that is, a fee for your health insurance whether you went to the doctor or not? About how much was it?
- Did you have to co-pays for doctor’s visits? About how much were they?
- And what about for prescriptions—how much did you pay for prescription medications?

1. Thinking about these costs taken together, describe for me how affordable they were for you.
2. Besides coverage for your doctors’ visits and medications, were there other benefits or services you received when had when you had Medicaid?

*Probe:*

- *If unclear what other benefits and services might include:* For example, home health care or dental care or vision care, or transportation to doctor’s appointments.

1. When you had Medicaid insurance, what did you like most about it?
2. When you had Medicaid insurance, what challenges (if any) did you have with it?

**Part 3. Navigating the Medicaid Expansion Coverage to Medicare Transition**

Now let’s talk about the process you went through to change your insurance and enroll in Medicare.

1. How did you learn that your Medicaid coverage was ending and you needed to enroll in Medicare?

*Probes:*

- Tell me about how you learned that you needed to sign up for Medicare—and that you would be losing your Medicaid expansion coverage?
- Did you get letters in the mail or phone calls about enrolling in Medicare and about your Medicaid coverage ending?
- Before you enrolled in Medicare, did you talk with friends or family members or someone else, such as a Medicare counselor about enrolling in Medicare? Tell me about those conversations.
- Did you enlist their help with enrolling in Medicare?

[Review screener survey responses]: We saw that you were/were not enrolled in X financial assistance program with Medicare costs…

1. When you were in the process of signing up for Medicare, what did you learn about programs that can provide financial help to reduce the cost of Medicare Part B premiums and copays for medications?  *Probe:*

- How did you learn about these programs that provided financial help? from website, a friend/relative, Medicare counselor?

**Part 4. Differences in Costs and Covered Benefits between Medicare and Medicaid Expansion Insurance**

Now I’d like to understand more about any differences you may have experienced between the Medicare coverage you have now compared to the Medicaid coverage you had before turning 65.

1. But first, I wanted to ask, **before** you enrolled in Medicare, how did you *think* Medicare would compare to the Medicaid coverage you had?
2. Ok, now in terms of actual differences you may have experienced let’s start with costs. Tell me about your health insurance and health care costs now since you enrolled in Medicare.
3. How do these costs compare to the health care costs you had when you had Medicaid?

*Probes:*

- Tell me about any differences between Medicaid coverage and Medicare in terms of how much you had to pay for the insurance itself?
- Tell me about any differences in what you pay now for doctor’s visits, medications compared to when you had Medicaid?

1. How affordable would you say are your Medicare costs for you?
2. Tell me about any ways in which the cost differences between Medicaid and Medicare have affected *how* you get health care.  *Probes:*

- Have there been any times since you signed up for Medicare where you had to delay getting or go without care or seeing a specific type of doctor because of the cost? Tell me about what that was like.
- *For those who indicated earlier in the interview they had enrolled in a Medicare Savings Program and/or Low-Income Subsidy to help defray cost of prescription drug costs, also called “Extra Help.”*

You mentioned earlier you were enrolled in [the name of program]. How helpful do you find the financial assistance it provides with premiums and/or copays with medications?

1. Have you experienced any differences in the types of services that your insurance now (Medicare) covers compared to Medicaid? Tell me about that. *Probes:*

- For example, did you have coverage for dental, vision, or hearing-related doctor’s visits when you had Medicaid?
- Have there been any changes in how you get transportation to doctors’ visits?
- Have there been any changes in how you get services at home (e.g., nursing, home health aide)?

1. Have you had to make any changes to your other spending in order to afford monthly payments or other costs for health care or medications? Tell me more about that.

**Part 5. Other Differences in Obtaining Health Care**

Now apart from costs, I’d like to understand how the change in your health insurance may have influenced how you go about getting health care.

1. Tell me about the experience of getting healthcare now that you have Medicare including whether are any differences compared to when you had Medicaid.  *Probes:*

- For example, do you see the same or different doctors than when you had Medicaid?
- Do you have a different co-pay?
- Are there any health care providers who you had access to under Medicaid who you no longer have access to under Medicare? Tell me about that and what it’s been like.
- And conversely, are there any health care providers you have access to now under Medicare that you didn’t before under Medicaid? Tell me about that and what it’s been like.
- Since you signed up for Medicare, has it been easy or difficult to find a provider who took your insurance or who was able to give you an appointment when you needed one?
- How satisfied are you with the care you receive from your health care providers and your relationship with them?

1. Thinking about these changes in the costs and in obtaining health care that we’ve been talking about it, how would you say these changes affected your health (if at all)?
2. What do you like (if anything) about your Medicare insurance?
3. Are there any challenges with your Medicare insurance that we haven’t discussed?

**Wrap-up and Closing**

Overall, would you say you have better or worse health care under Medicare than when you had Medicaid?

Lastly, if you were advising a friend who was making the transition from the Healthy Michigan Plan to Medicare, what advice would you give them?

*Probe:*

*If unclear what we’re asking*--in other words, is there anything you wish you had known beforehand about the transition from Medicaid to Medicare?

That’s the end of the interview. Thank you very much for taking the time to share your experiences and thoughts with us!

**References**

1. **Burns, A, Mohamed, M, & Watts, MO**. *Medicaid-eligibility levels for older adults and people with disabilities (non-MAGI).* Kaiser Family Foundation. April 7, 2025. Available at: <https://www.kff.org/medicaid/issue-brief/medicaid-eligibility-levels-for-older-adults-and-people-with-disabilities-non-magi-in-2025/>. Accessed July 31, 2025.
2. **California Department of Health Care Services**. Income disregard for the aged, blind & disabled federal poverty level program. DHCS. November 23, 2020. Available at: <https://www.dhcs.ca.gov/services/medi-cal/eligibility/letters/Documents/20-24.pdf>. Accessed July 31, 2025.
3. **New York State Department of Health**. New York State Income and Resource Standards for Non-MAGI Population. NYSDOH. January 1, 2025. Available at: <https://www.health.ny.gov/health_care/medicaid/publications/docs/gis/25ma01_att1.pdf>. Accessed July 31, 2025.
4. **California Department of Health Care Services**. Aged Asset Limit Changes for Non-MAGI Medi-Cal. DHCS. January 1, 2024. Available at: <https://www.dhcs.ca.gov/services/medi-cal/eligibility/Pages/Asset-Limit-Changes-for-Non-MAGI-Medi-Cal.aspx>. Accessed July 31, 2025.
5. **Michigan Department of Health & Human Services**. Healthy Michigan Plan Progress Report. MDHHS. May 27, 2025. Available at: <https://www.michigan.gov/mdhhs/assistance-programs/healthcare/healthymichigan>. Accessed July 31, 2025.
